# Acute *Streptococcus mitis* Sacroiliitis in a Teenager with Unclear Source of Bacteremia: A Case Report and Literature Review

**DOI:** 10.1155/2018/2616787

**Published:** 2018-10-09

**Authors:** Fatma Al-Farsi, Ibrahim Al-Busaidi, Khalfan Al-Zeedi

**Affiliations:** ^1^Oman Medical Specialty Board, Medical Microbiology Residency Program, Muscat, Oman; ^2^Infectious Diseases Unit, Department of Medicine, Sultan Qaboos University Hospital, Muscat, Oman; ^3^Department of Medicine, Sultan Qaboos University Hospital, Muscat, Oman

## Abstract

Septic arthritis is an orthopedic emergency that is commonly caused by *Staphylococcus aureus*. Old age, diabetes mellitus, rheumatoid arthritis, prosthetic joint, and recent surgery are the main predisposing risk factors. Most cases of septic arthritis are caused by hematogenous spread of infection. Infectious sacroiliitis is a rare form of septic arthritis which is often clinically challenging to diagnose due its various and nonspecific presentations. *Streptococcus mitis* belongs to viridans group streptococci (VGS) bacteria, which is a component of body flora that is commonly involved in bacterial endocarditis. VGS in general and *S. mitis* specifically is an uncommon cause of osteoarticular infections. Here, we report a case of spontaneous *Streptococcus mitis* bacteremia complicated by septic sacroiliitis in a healthy teenager in the absence of infective endocarditis or a clear source of bacteremia.

## 1. Introduction

Septic arthritis more commonly involves large joints than small joints, and in up to 60% of cases, the hip or the knee is involved [1, 2]. Septic arthritis of the sacroiliac joint is rare, and it represents only 1-2% of all cases of septic arthritis [3]. The predominant causative pathogens in septic arthritis are *Staphylococcus aureus* and *β*-hemolytic *Streptococcus*, accounting for up to 91% of cases. In the elderly and the immunocompromised hosts, infection with a Gram-negative bacillus is more common. Septic arthritis due to anaerobic organisms complicating penetrating trauma has been reported [4].

*Streptococcus mitis* is an alpha-hemolytic species belonging to the family of viridans group streptococci (VGS)*. S. mitis* is a component of the normal oropharynx, skin, and gastrointestinal and genital tract floras [5]. VGS are the second most common causative organisms of bacterial endocarditis, but they are rarely associated with septic arthritis. Furthermore, *Streptococcus mitis*, a subgroup of VGS, has been implicated even less commonly [6]. *S. mitis* septic arthritis has been rarely reported in the literature in association with poor dentition and following dental procedures. In this article, we report a rare case of spontaneous septic sacroiliitis complicating *S. mitis* bacteremia in a healthy young man.

## 2. Case Report

A 19-year-old previously healthy teenager presented to the emergency room with acute severe left gluteal and posterior thigh pain for one-day duration associated with restricted hip movement and inability to bear weight on his left lower limb. He had no other joints' involvement. He also had fever for one-day duration without chills or rigors. He had no recent intra-articular or intravenous injection or any trauma. A review of systems was negative including a recent diarrhea illness, urinary tract infection, or urethritis. He was not a smoker, an alcohol consumer, or an IV drug abuser. He had no recent sexual activity or any previous history of sexually transmitted infection. At triaging in the emergency department, he was alert and oriented but looked in pain. He was febrile, temperature (38.0°C), but otherwise he had stable vitals. Physical examination revealed diffuse tenderness in the left gluteal region and left hip joint with restricted internal and external rotation and extension of the left hip, and the FABER test was positive. There were no skin changes overlying the hip. Initial plain X-ray and CT scan of the hip showed no abnormalities.

Laboratory investigations showed mild neutrophilic leukocytosis with total white cell count (11.4 × 10^9^ per liter). His serum C-reactive protein (CRP) was elevated (56 mg/dL) with normal erythrocyte sedimentation rate (ESR) (11 mm/h). Septic arthritis of the hip joint was suspected, and he was started empirically on intravenous vancomycin and ceftriaxone 2 g daily.

**Figure 1:**
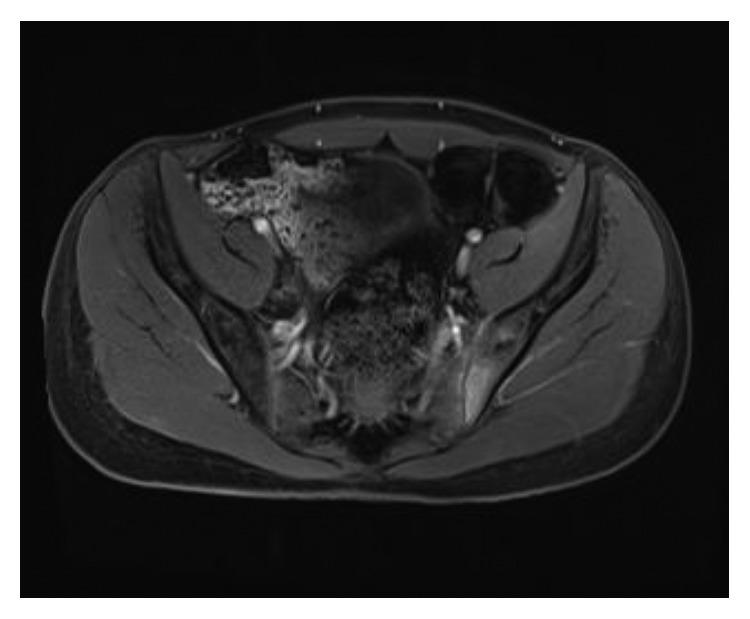
MRI of the hip showing axial T1-weighted postcontrast enhancement involving the visualized parts of the left ilium and sacrum at the sacroiliac joint.

On his third day of admission, blood culture grew penicillin-susceptible *Streptococcus mitis*. Magnetic resonance imaging (MRI) of the left hip showed features of left-sided sacroiliitis with significant subchondral edema with minimal amount of free fluid along the inferior aspects of the left sacroiliac joint (Figure 1). The synovial fluid was too small to drain, and the patient already started to improve with intravenous therapy. The clinical presentations and hip MRI findings confirmed the diagnosis of septic sacroiliitis due to *Streptococcus mitis*. Transthoracic echocardiogram showed no valvular abnormality or vegetation. Subsequent blood cultures were negative. He was continued on IV ceftriaxone (1 g once daily) for a total of four weeks. On follow-up, the patient slowly improved, and at the completion of his antibiotic therapy, he became pain free and he was able to bear weight and walk normally. Both WBC and CRP normalized at the end of the antibiotic course.

## 3. Discussion

Septic arthritis of the sacroiliac joint is rare, and it represents only 1-2% of total cases of septic arthritis [7]. Lower back pain, lumbogluteal pain, hip pain, and/or posterior thigh pain, with movement restriction of the affected side, are the most common reported symptoms. In our patient, other causes of acute unilateral sacroiliitis were considered including brucellosis, tuberculosis, ankylosing spondylitis, and reactive or seronegative sacroiliitis. The workup for all of these causes including *Brucella* serology and autoimmune workup was negative.

Due to nonspecific clinical presentation, diagnosis of septic sacroiliitis can be time-consuming and clinically challenging. In our patient, fever and positive blood culture support an infectious cause of his unilateral sacroiliitis. Delayed diagnosis results in an increased risk for permanent bone destruction or septicemia [8]. Infection by the *Streptococcus viridans* group usually occurs in a previously injured focus; however, its association with dental caries and bacterial endocarditis is well established [9]. Septic arthritis caused by *S. viridans* predominantly involving the knee joint has been reported in patients with severe osteoarthritis, poor dental hygiene, and intravenous drug use [9–11].

*S. mitis*, a member of the viridans group streptococci, is a very rare cause of sacroiliitis. To our knowledge, this is the first reported case of *S. mitis* sacroiliitis. Septic arthritis caused by this organism was reported to occur as a complication of hematogenous seeding of the sacroiliac joint in patients with infective endocarditis [12]. Thus, echocardiography (ECHO) is essential to rule out endocarditis in the setting of complicated viridans group streptococci bacteremia [13]. In our case, ECHO did not show any evidence of endocarditis. The primary focus of *S. mitis* bacteremia is usually the oral cavity due to poor dentition or mucosal injury or gastrointestinal tract. Oren and colleagues reported cases of *S. mitis* septic arthritis of the glenohumeral joint that likely resulted from hematogenous spread after oral trauma in a patient with poor dentition [6]. However, in our patient, there was no apparent primary focus or point of entry of *S. mitis*. Cariati and Deng described a case of thoracic spondylodiscitis caused by *S. mitis* in a patient with chronic sinusitis [14]. Three recent case studies reported absence of apparent primary infection or port of entry of organism including a child with *S. mitis* bacteremia and osteomyelitis [15], a 57-year-old woman with septic arthritis of pubic symphysis [16], and a 49-year-old man with spondylodiscitis [17].

MRI has become the imaging modality of choice for the diagnosis of infectious sacroiliitis [18]. As illustrated in our case, signs of infection on CT scan can be absent in the early stage of septic sacroiliitis. Our patient demonstrated typical MRI findings of sacroiliitis.

Prompt treatment with intravenous antibiotics together and drainage of any purulent material are the mainstay treatment for septic arthritis [1]. Empirical antibiotic therapy should have anti-*Staphylococcus aureus* cover until the causative organism is identified and antibiotic susceptibility profile is reported [18]. Our patient was empirically started on intravenous vancomycin and ceftriaxone and then switched to intravenous ceftriaxone based on the blood cultures and susceptibility results of the isolated *S. mitis*. Surgical drainage and washout of the infected sacroiliac joint is rarely considered in cases with severe infection or poor clinical response to antibiotic therapy. Our patient had immediate and satisfactory response to intravenous antibiotic therapy, and no surgical intervention was needed.

## 4. Conclusion

Isolated infection of the sacroiliac joint is rare and therefore frequently misdiagnosed. *S. mitis*, a member of the viridans group streptococci, is a rare cause of septic sacroiliitis. We have reported a case of *S. mitis* bacteremia and septic sacroiliitis in the absence of an obvious primary focus in a young healthy teenager. MRI should be considered in cases where septic sacroiliitis is highly suspected despite normal plain X-rays and CT scan. Echocardiography is recommended in complicated cases of *S. mitis* bacteremia to rule out endocarditis. Early and targeted antibiotic therapy with or without surgical drainage or washout is essential in the management of septic sacroiliitis.
